# Age- and sex-dependency of thrombin generation parameters in the general Italian population: the Moli-sani study

**DOI:** 10.3389/fcvm.2025.1528871

**Published:** 2025-02-21

**Authors:** Simona Costanzo, Bas de Laat, Augusto Di Castelnuovo, Lisa van der Vorm, Amalia De Curtis, Chiara Cerletti, Marisa Ninivaggi, Maria Benedetta Donati, Licia Iacoviello, Romy de Laat-Kremers, the Moli-sani investigators, Licia Iacoviello

**Affiliations:** ^1^ Research Unit of Epidemiology and Prevention, IRCCS Neuromed, Pozzilli, Italy; ^2^ Department of Data Analysis and Artificial Intelligence, Synapse Research Institute, Maastricht, Netherlands; ^3^ Department of Functional Coagulation, Synapse Research Institute, Maastricht, Netherlands; ^4^ Department of Medicine and Surgery, LUM University “Giuseppe Degennaro”, Casamassima, Italy

**Keywords:** age, sex, coagulation, thrombin, thrombin generation, thrombomodulin

## Abstract

**Background:**

Recent developments have made the thrombin generation (TG) test accessible to the clinical laboratory. Therefore, the clinical interpretation of TG parameters has become of increasing interest, and reference values are required. Age and sex have been shown to affect TG parameters, but no consensus has been reached on the subject. We investigated the effect of age and sex on TG parameters to determine the need for age and sex specific reference values for TG.

**Methods:**

TG was measured in 22,014 individuals of the Moli-sani study, a cross-sectional cohort of the general adult Italian population. TG was measured using Calibrated Automated Thrombinography in platelet poor plasma (PPP) using PPP reagent Low and PPP reagent, in the presence and absence of thrombomodulin (TM).

**Results:**

Individuals were grouped into 6 age categories: 35–44 years of age (*n* = 5,073), 45–54 (*n* = 6,448), 55–64 (*n* = 5,516), 65–74 (*n* = 3,539), 75–84 (*n* = 1,261), and 85 years of age and older (*n* = 106). Men and women were distributed evenly in the age categories. Smoking was more common at younger age, whereas cardiovascular diseases, hypertension, hypercholesterolemia, and diabetes were more common at older age (*p* < 0.001). Lag time and velocity index increased with age, whereas the endogenous thrombin potential (ETP) and time-to-peak decreased. The inhibitory effect of TM was reduced at higher age (*p* < 0.001). The TG lag time was shorter in women than men at younger age (6%–7% lower in women), and the ETP was lower in women. The activated protein C (APC) pathway was desensitized in women and older individuals.

**Conclusion:**

The TG profile becomes more “procoagulant” at older age, especially in women. The sensitivity of the APC pathway to TM is reduced with increasing age in men and women. Therefore, age and sex appropriate reference values for TG parameters would be of interest for the ongoing clinical implementation of the TG assay.

## Introduction

The thrombin generation (TG) test is a global hemostasis test (1). High TG has been reported to be associated with thrombotic events (2–4), whereas low TG has been related to bleeding symptoms in patients with bleeding disorders (5–9) and during surgery (10). Recent developments of fully automated analyzers for TG have made the test more easily accessible in the clinical laboratory (11, 12). As a result, the clinical interpretation of TG parameters and their association with specific outcomes has become of increasing interest. Reference values for TG have been established to allow the clinical interpretation of TG results (13, 14). Typically, reference values are determined in 120 healthy individuals between 18 and 65 years of age. As a consequence, published reference values for TG are based on healthy adults up to 65, although the risk of (non-hereditary) thrombosis is known to increase with age (15–18). Age has been shown to affect TG under various circumstances (19–23), but a consensus on the effect of age on TG has not yet been established. In the Gutenberg Health Study (*n* = 4,843), older age was associated with a longer lag time (19). Peak height and the endogenous thrombin potential (ETP) increased with age in men, but not in women (21).

The risk of thrombosis is known to be sex-dependent (24). This association can be partially explained by clinical risk factors that are predominantly present in one of both sexes, such as pregnancy, use of oral contraceptives (OCs), hormone replacement therapy, and estrogen-antagonist therapies in women (18), and higher body height (on average) in men (25). Women have been shown to have a higher TG peak height than men (20). When comparing men to women without oral contraceptives (OCs), no differences in TG were found (26). However, women using OCs are known to have a more procoagulant TG profile compared to both men and women without OCs (26). Typically, peak height and ETP are higher in women using OCs (27). If thrombomodulin (TM) is present during the measurements, the effect of OCs use becomes more pronounced (28). TM is a vessel wall derived protein that forms a complex with thrombin and subsequently acts as an activator of protein C. Activated protein C (APC) is a direct inhibitor of procoagulant co-factors V and VIII. Via TM and the APC pathway, thrombin can downregulate its own production during a coagulation response (29, 30). A dedicated kit has been developed for the TG test, in which the sensitivity of a subject's coagulation system is assessed via the addition of TM (2, 31). Women using OCs are less sensitive to the inhibitory actions of TM in TG (2), and certain pathological states such as the antiphospholipid syndrome, and the Factor V Leiden mutation, are known to lead to APC resistance in TG (32, 33).

Because of the age- and sex-dependency of the thrombosis risk, it is of interest to compare a patients’ TG result to an age-appropriate reference population, as this might have direct implications for their clinical interpretation. In the current study, we aimed to study the association of age, sex, and TG parameters in 24,325 individuals of the Moli-sani cohort, from a general Italian adult population.

## Methods

### Subjects and samples

The Moli-sani cohort participants were randomly recruited in the Molise region in Italy using city hall registries, as previously described, after providing written informed consent (34, 35). The study was approved by the ethics committee of the Catholic University of Rome (Italy) and complies with the Declaration of Helsinki. Blood samples were obtained by venipuncture from participants who had fasted overnight and had refrained from smoking for at least 6 h (35). Platelet poor plasma was prepared by centrifuging twice at 2,821 g for 10 min, and citrated plasma samples were stored in liquid nitrogen at the Neuromed Biobanking Center until shipment to the Synapse laboratory, where they were stored at −80°C until further analysis. The levels of labile coagulation factors FV and FVIII were determined in a subset of 144 samples to confirm plasma sample quality.

### Selection of data for analysis

**Figure 1:**
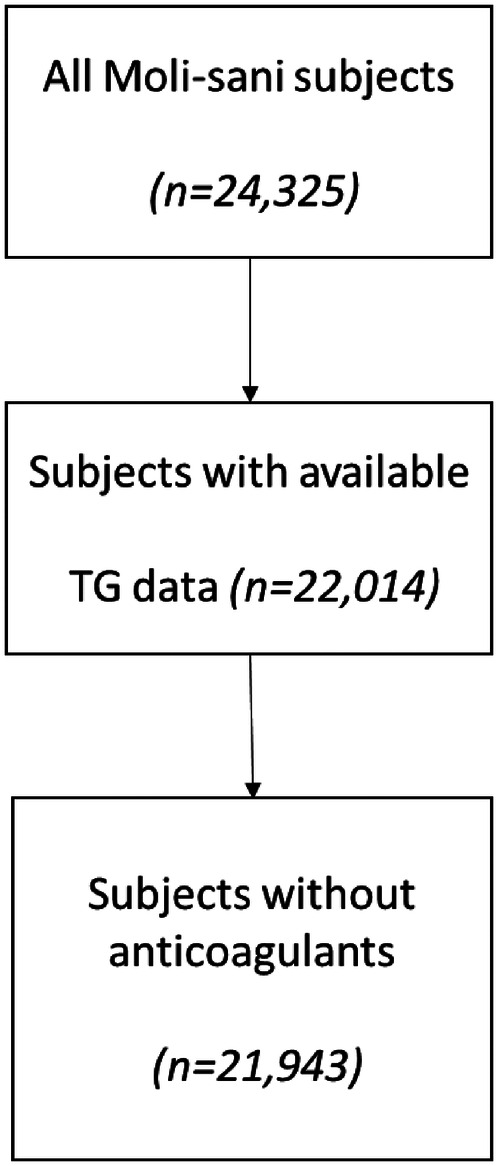
Flow chart for sample selection and analysis.

Exclusion criteria for the Moli-sani study were age below 35, pregnancy at the time of recruitment, disturbances in understanding or willingness, current poly-traumas or coma, or refusal to sign the informed consent. In the Moli-sani cohort, 24,325 individuals were enrolled. For thrombin generation-based analysis subjects with missing TG data and subjects using medication known to affect TG (Vitamin K antagonists or heparins) were excluded from analysis. Due to the time of the baseline sample collection of the Moli-sani samples (2005–2010), no subjects reported the use of DOACs at baseline. Sufficient plasma volume was available to measure TG in 22,014 samples (Figure 1). Subjects using anticoagulants (vitamin K antagonists or heparins) were excluded from analysis due to their known effect on TG, leaving TG data from 21,943 individuals for statistical analysis.

### Baseline data collection

At the baseline visit questionnaires were filled out to obtain information on socioeconomic status, physical activity, medical history, dietary habits, risk factors, personal and family medical history, and drug use. The questionnaire on drug use was directly linked to the Italian National drug index. Use of vitamin K antagonists, antiplatelet medication, heparin, and oral contraceptives was recorded. Body Mass Index (BMI) was calculated as weight/(height)^2^ (kg/m^2^). Blood pressure was measured at time of inclusion and blood sampling, by an automatic device (OMRON-HEM-705CP) three times on the non-dominant arm, with the patient lying down for about 5 min, and the average of the last two values was taken as the BP. Hypertension was defined as systolic BP (SBP) ≥ 140 mmHg or diastolic BP (DBP) ≥ 90 mmHg or based on current treatment with antihypertensive drugs. Pre-hypertension was defined as SBP of 130–139 mmHg or DBP of 85–89 mmHg.

History of CVD (including angina, acute myocardial infarction, revascularization procedures and cerebrovascular events and peripheral artery disease) was self-reported and confirmed by medical records. Serum lipids (HDL-cholesterol, triglycerides) and blood glucose were assayed by enzymatic reaction methods using an automatic analyzer [ILab 350, Instrumentation Laboratory (IL), Milan, Italy]. Hypercholesterolemia was defined as serum total cholesterol ≥240 mg/dl or current treatment with Cholesterol Medications. Pre-hypercholesterolemia was defined as serum total cholesterol of 200–239 mg/dl. Diabetes was defined as serum glucose ≥126 mg/dl or based on current treatment with Diabetes Medications. Pre-diabetes was defined as serum glucose of 110–125 mg/dl.

### Thrombin generation

TG was measured using Calibrated Automated Thrombinography (CAT) using platelet poor plasma (PPP) reagent Low and PPP reagent, in the presence and absence of TM (2). PPP Reagent, PPP Reagent Low, Thrombin Calibrator and FluCa kits were used in accordance with the manufacturer's recommendations (Diagnostica Stago, France). Data were analyzed with the CAT-associated Thrombinoscope software, and TG parameter values were exported for further analysis (36). The sensitivity of the activated protein C pathway was tested through the addition of TM. The inhibitory effect of TM was quantified as the inhibition of the ETP in the presence of TM compared with TG triggered with PPP Reagent without added TM.

### Statistics

Spearman's correlation coefficient was used for initial correlations between TG and age. For all further analyses, the study population was divided into age categories to enable the comparison of clinical data from patient studies to the values found in the general population, according to the appropriate age. The minimum inclusion age of the Moli-sani study is 35 years, and therefore the following age categories were selected: 35–44 years of age, 45–54 years of age, 55–64 years of age, 65–74 years of age, 75–84 years of age, and 85 years of age and older. TG parameters were not normally distributed according to the Kolmogorov–Smirnov test. Subsequently, differences in continuous variables between age groups were analyzed by the Kruskal–Wallis test, whereas differences in categorical variables were determined by the Chi-Square test.

## Results

In total, TG data of 22,014 samples of the Moli-sani cohort were available for statistical analysis. Seventy-five individuals were excluded from analysis due to anticoagulant use, which affects TG. Forty-eight percent of the subjects was male, and the average age was 55.3 ± 11.7 years. The subjects (*n* = 21,943) were grouped into 6 age categories: 35–44 years of age (*n* = 5,073), 45–54 (*n* = 6,448), 55–64 (*n* = 5,516), 65–74 (*n* = 3,539), 75–84 (*n* = 1,261), and 85 years of age and older (*n* = 106). Age was 41, 50, 60, 70, 79 and 88 years on average, respectively for the defined age categories (Table 1). Women were slightly overrepresented in lower age categories (54%), whereas in higher age categories, sex was evenly distributed fifty-fifty between men and women. Smoking was more common at younger age, whereas cardiovascular diseases, hypertension, hypercholesterolemia, and diabetes were more common at older age (*p* < 0.001). For women, OC use was higher in lower age categories, as expected (*p* < 0.001).

**Table 1:** General characteristics of the Moli-sani cohort, stratified for age and sex.

Age (years)	35–44	45–54	55–64	65–74	75–84	85+	*p*-value
All subjects							
*N*	5,073	6,448	5,516	3,539	1,261	106	
Age	41.0 ± 2.5	50.0 ± 2.9	59.6 ± 2.8	69.6 ± 2.8	78.6 ± 2.7	88.1 ± 2.9	<0.001
Sex (% male)	2,317 (46%)	2,934 (46%)	2,754 (50%)	1,803 (51%)	637 (51%)	53 (50%)	<0.001
BMI	26.5 ± 4.6	27.8 ± 4.6	28.9 ± 4.7	29.0 ± 4.7	28.6 ± 4.6	27.1 ± 4.5	<0.001
Smoking	1,488 (29%)	1,891 (29%)	1,196 (22%)	428 (12%)	90 (7%)	2 (2%)	<0.001
Cardiovascular diseases	27 (1%)	130 (2%)	318 (6%)	452 (13%)	230 (18%)	20 (19%)	<0.001
Hypertension	2,205 (43%)	4,247 (66%)	4,588 (83%)	3,257 (92%)	1,198 (95%)	102 (96%)	<0.001
Hypercholesterolemia	2,595 (51%)	4,324 (67%)	3,956 (72%)	2,420 (68%)	794 (63%)	58 (55%)	<0.001
Diabetes	446 (9%)	1,121 (17%)	1,475 (27%)	1,074 (30%)	393 (31%)	30 (28%)	<0.001
Men							
*N*	2,317	2,934	2,754	1,803	637	53	
Age	41.0 ± 2.5	50.0 ± 0.0	59.7 ± 2.8	69.6 ± 2.8	78.6 ± 2.6	88.1 ± 3.3	<0.001
BMI	27.5 ± 4.0	28.3 ± 4.0	28.7 ± 4.0	28.5 ± 4.0	27.9 ± 4.1	26.2 ± 3.5	<0.001
Smoking	794 (34%)	911 (31%)	681 (25%)	280 (16%)	70 (11%)	2 (4%)	<0.001
Cardiovascular diseases	15 (1%)	82 (3%)	209 (8%)	312 (17%)	150 (24%)	10 (19%)	<0.001
Hypertension	1,381 (60%)	2,198 (75%)	2,382 (86%)	1,763 (98%)	601 (94%)	50 (94%)	<0.001
Hypercholesterolemia	1,334 (58%)	1,967 (67%)	1,807 (66%)	1,087 (60%)	330 (52%)	26 (49%)	<0.001
Diabetes	326 (14%)	753 (26%)	955 (35%)	670 (37%)	242 (38%)	18 (34%)	<0.001
Women							
*N*	2,756	3,514	2,762	1,736	624	53	
Age	41.0 ± 2.5	50.0 ± 2.9	59.6 ± 2.8	69.6 ± 2.8	78.6 ± 2.7	88.1 ± 2.5	<0.001
BMI	25.6 ± 4.9	27.4 ± 5.1	29.2 ± 5.3	29.6 ± 5.2	29.3 ± 5.0	28.2 ± 5.1	<0.001
Smoking	694 (25%)	980 (28%)	515 (19%)	148 (9%)	20 (3%)	0 (0%)	<0.001
Cardiovascular diseases	12 (<1%)	48 (1%)	109 (4%)	140 (8%)	80 (13%)	10 (19%)	<0.001
Hypertension	824 (30%)	2,049 (58%)	2,206 (80%	1,584 (91%)	597 (96%)	52 (98%)	<0.001
Hypercholesterolemia	1,261 (46%)	2,357 (67%)	2,149 (78%)	1,333 (77%)	464 (74%)	32 (60%)	<0.001
Diabetes	120 (4%)	368 (10)	520 (19%)	404 (23%)	151 (24%)	12 (23%)	<0.001
OCs use	421 (15%)	325 (9%)	94 (3%)	16 (1%)	1 (0%)	1 (2%)	<0.001

OCs, oral contraceptives; BMI, body mass index.

### Thrombin generation

TG was measured using PPP Reagent Low and PPP Reagent, and the sensitivity of TG to TM was determined. Lag time and velocity index increased with age, both when TG was triggered using PPP Reagent Low and PPP Reagent (Table 2; *p* < 0.001). ETP and time-to-peak decreased with age for both trigger reagents (*p* < 0.001). When TG was triggered with PPP Reagent, the peak height was higher in older individuals (*p* < 0.001). Additionally, the inhibitory effect of TM was reduced at higher age (*p* < 0.001).

**Table 2:** Spearman's correlation of thrombin generation parameters with age, stratified for sex.

	All subjects	*p*-value	Men	*p*-value	Women	*p*-value
PPP reagent low						
Lag time (min)	0.026	*<0*.*001*	−0.060	*<0*.*001*	0.118	*<0*.*001*
ETP (nM•min)	−0.089	*<0*.*001*	−0.105	*<0*.*001*	−0.085	*<0*.*001*
Peak (nM)	0.011	*0*.*109*	−0.036	*<0*.*001*	0.045	*<0*.*001*
Time-to-peak (min)	−0.046	*<0*.*001*	−0.110	*<0*.*001*	0.021	*0*.*024*
Velocity index (nM/min)	0.087	*<0*.*001*	0.068	*<0*.*001*	0.098	*<0*.*001*
PPP reagent						
Lag time (min)	0.037	*<0*.*001*	−0.059	*<0*.*001*	0.139	*<0*.*001*
ETP (nM•min)	−0.086	*<0*.*001*	−0.102	*<0*.*001*	−0.080	*<0*.*001*
Peak (nM)	−0.015	*0*.*024*	−0.039	*<0*.*001*	0.002	*0*.*863*
Time-to-peak (min)	−0.041	*<0*.*001*	−0.131	*<0*.*001*	0.049	*<0*.*001*
Velocity index (nM/min)	0.055	*<0*.*001*	0.072	*<0*.*001*	0.038	*<0*.*001*
Thrombomodulin						
ETP inhibition by TM (%)	−0.135	*<0*.*001*	−0.224	*<0*.*001*	−0.052	*<0*.*001*

ETP, endogenous thrombin potential; PPP, platelet poor plasma.

Italic values denote correlations were quantified by Spearman's Rho and *p* values below 0.05 were considered statistically significant.

### Effect of age and sex on thrombin generation parameters

We further analyzed the association of age and TG parameters using multivariate regression analysis (Table 3). The association of age TG parameters followed similar patterns for both the trigger condition with a low concentration of tissue factor (TF; PPP Reagent Low) and a regular TF concentration (PP Reagent). ETP significantly declined in both men and women with increasing age, even after correction for life style factors and medical history. Peak height decreased significantly with age in men in the fully adjusted model, but not in women. The velocity index increases with increasing age, albeit more pronounced in men than women. Interestingly, clear differences between men and women were found for the association of time-dependent TG parameters and age. TG lag time and time-to-peak were significantly prolonged with increasing age in women, but were shorter in older than younger men.

**Table 3:** Multivariable regression analysis of thrombin generation parameters and age.

		Lag time (min)			ETP (nM•min)			Peak (nM)			Time-to-peak (min)			Velocity index (nM/min)		
		B	95% CI	*p*-value	B	95% CI	*p*-value	B	95% CI	*p*-value	B	95% CI	*p*-value	B	95% CI	*p*-value
PPP reagent low																
All subjects	Model 1	0.004	0.003–0.005	<0.001	−3.758	−4.220 to −3.296	<0.001	−0.090	−0.190 to 0.010	0.077	−0.004	−0.006 to −0.003	<0.001	0.421	0.351–0.492	<0.001
	Model 2	0.004	0.002–0.005	<0.001	−3.628	−4.088 to −3.167	<0.001	−0.063	−0.163 to 0.036	0.214	−0.005	−0.006 to −0.003	<0.001	0.433	0.362–0.504	<0.001
	Model 3	0.002	0.001–0.003	<0.001	−4.709	−5.176 to −4.242	<0.001	−0.269	−0.370 to −0.167	<0.001	−0.006	−0.007 to −0.004	<0.001	0.317	0.245–0.390	<0.001
	Model 4	0.001	0.000–0.003	0.031	−4.731	−5.242 to −4.22	<0.001	−0.434	−0.546 to −0.322	<0.001	−0.005	−0.007 to −0.003	<0.001	0.191	0.111–0.271	<0.001
Men	Model 1	−0.002	−0.004 to −0.001	0.008	−3.743	−4.387 to −3.098	<0.001	−0.351	−0.491 to −0.211	<0.001	−0.011	−0.014 to −0.009	<0.001	0.328	0.229–0.426	<0.001
	Model 2	n/a	n/a	n/a	n/a	n/a	n/a	n/a	n/a	n/a	n/a	n/a	n/a	n/a	n/a	n/a
	Model 3	−0.002	−0.004 to −0.001	<0.001	−3.797	−4.446 to −3.148	<0.001	−0.396	−0.538 to −0.254	<0.001	−0.011	−0.014 to −0.008	<0.001	0.291	0.190–0.391	<0.001
	Model 4	−0.002	−0.004 to 0.000	0.037	−3.000	−3.699 to −2.302	<0.001	−0.410	−0.563 to −0.256	<0.001	−0.009	−0.012 to −0.006	<0.001	0.214	0.105–0.323	<0.001
Women	Model 1	0.009	0.008–0.011	<0.001	−3.516	−4.174 to −2.858	<0.001	0.214	0.072–0.356	0.003	0.002	−0.001 to 0.004	0.15	0.535	0.434–0.637	<0.001
	Model 2	n/a	n/a	n/a	n/a	n/a	n/a	n/a	n/a	n/a	n/a	n/a	n/a	n/a	n/a	n/a
	Model 3	0.007	0.005–0.008	<0.001	−5.720	−6.393 to −5.046	<0.001	−0.155	−0.302 to −0.008	0.038	0.000	−0.002 to 0.002	0.994	0.338	0.233–0.444	<0.001
	Model 4	0.005	0.003–0.007	<0.001	−6.788	−7.549 to −6.027	<0.001	−0.490	−0.657 to −0.323	<0.001	−0.001	−0.003 to 0.002	0.697	0.158	0.037–0.278	0.01
PPP Reagent																
All subjects	Model 1	0.003	0.002–0.004	<0.001	−3.696	−4.174 to −3.219	<0.001	−0.274	−0.367 to −0.181	<0.001	−0.002	−0.004 to −0.001	<0.001	0.231	0.167–0.294	<0.001
	Model 2	0.003	0.002–0.004	<0.001	−3.569	−4.045 to −3.092	<0.001	−0.238	−0.331 to −0.146	<0.001	−0.003	−0.004 to −0.002	<0.001	0.251	0.188–0.314	<0.001
	Model 3	0.002	0.001–0.003	<0.001	−4.668	−5.151 to −4.185	<0.001	−0.421	−0.515 to −0.326	<0.001	−0.003	−0.005 to −0.002	<0.001	0.157	0.092–0.221	<0.001
	Model 4	0.001	0.000–0.002	0.002	−4.658	−5.187 to −4.128	<0.001	−0.531	−0.636 to −0.427	<0.001	−0.003	−0.005 to −0.002	<0.001	0.073	0.002–0.145	0.045
Men	Model 1	−0.001	−0.002–0.000	0.08	−3.690	−4.355 to −3.025	<0.001	−0.363	−0.492 to −0.233	<0.001	−0.009	−0.011 to −0.007	<0.001	0.311	0.223–0.399	<0.001
	Model 2	n/a	n/a	n/a	n/a	n/a	n/a	n/a	n/a	n/a	n/a	n/a	n/a	n/a	n/a	n/a
	Model 3	−0.001	−0.002 to 0.000	<0.001	−3.767	−4.437 to −3.097	<0.001	−0.408	−0.539 to −0.276	<0.001	−0.009	−0.011 to −0.007	<0.001	0.274	0.185–0.363	<0.001
	Model 4	−0.001	−0.002 to 0.000	0.111	−2.931	−3.653 to −2.210	<0.001	−0.394	−0.536 to −0.252	<0.001	−0.007	−0.009 to −0.005	<0.001	0.210	0.113–0.307	<0.001
Women	Model 1	0.007	0.006–0.007	<0.001	−3.452	−4.134 to −2.771	<0.001	−0.119	−0.251 to 0.013	0.078	0.003	0.002–0.005	<0.001	0.194	0.103–0.284	<0.001
	Model 2	n/a	n/a	n/a	n/a	n/a	n/a	n/a	n/a	n/a	n/a	n/a	n/a	n/a	n/a	n/a
	Model 3	0.005	0.004–0.006	<0.001	−5.657	−6.356 to −4.958	<0.001	−0.450	−0.586 to −0.313	<0.001	0.002	0.000–0.003	0.018	0.032	−0.062 to 0.126	0.505
	Model 4	0.004	0.003–0.005	<0.001	−6.699	−7.489 to −5.908	<0.001	−0.712	−0.868 to −0.557	<0.001	0.001	−0.001 to 0.002	0.529	−0.082	−0.189 to 0.026	0.135

Model 1 was the crude model based on the association of age with each TG parameter; Model 2* is adjusted for sex; Model 3 is adjusted for sex, BMI, and smoking; Model 4 is adjusted sex, body mass index, smoking, and medical history (cardiovascular diseases, hypertension, hypercholesterolemia, and diabetes). *Model 2 was not applicable in the sub-cohorts of men and women, as sex could not be corrected for in the sex-based sub-cohorts. BMI, body mass index; CI, confidence interval; ETP, endogenous thrombin potential; PPP, platelet poor plasma.

**Figure 2:**
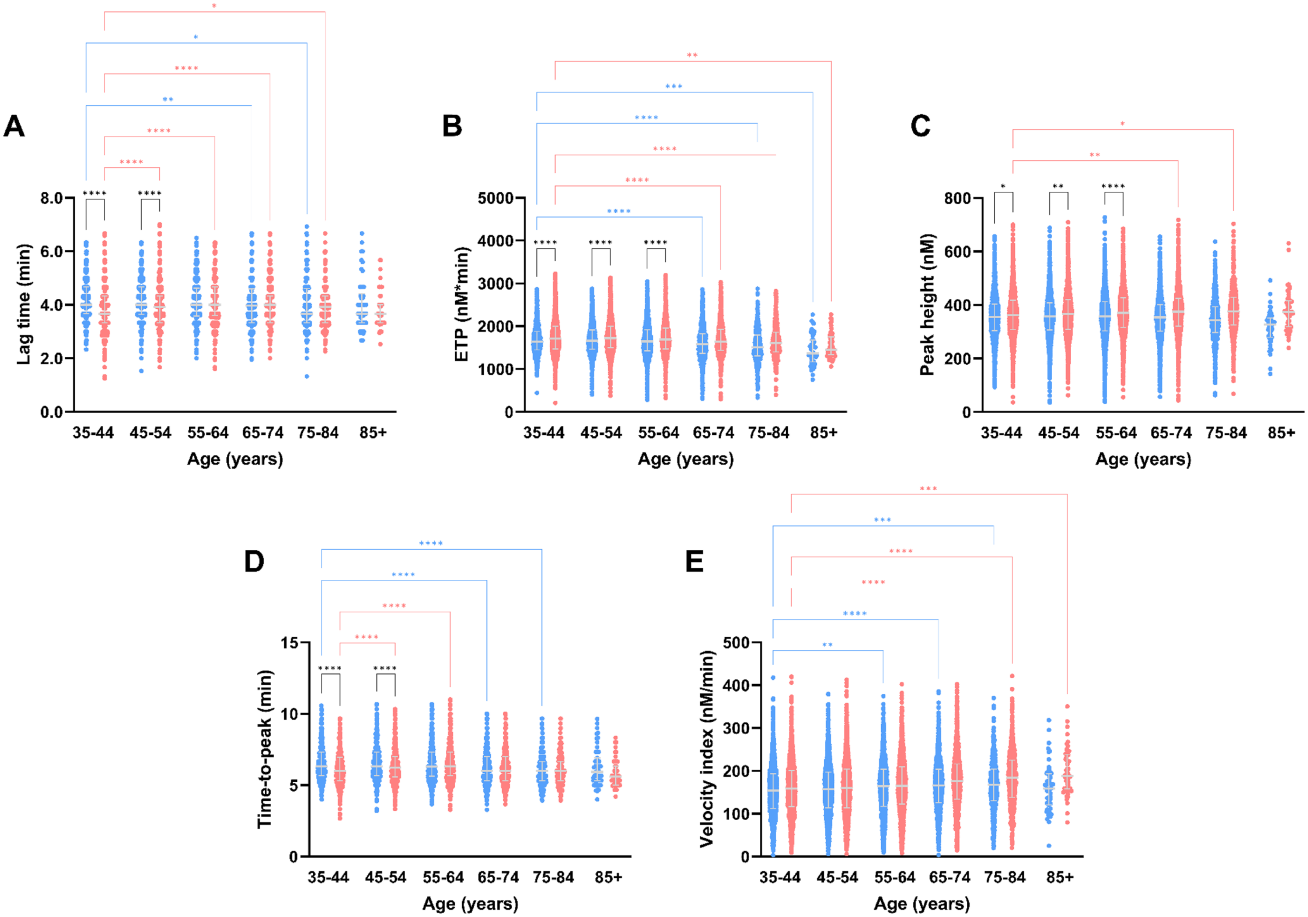
Thrombin generation parameters measured after stimulation with PPP reagent Low, stratified for age and sex. TG parameters lag time **(A)**, peak height **(B)**, time-to-peak **(C)**, ETP **(D)** and velocity index **(E)** according to the different age categories in the Moli-sani cohort. Data are depicted as the median and interquartile range. Data was stratified for sex, showing the data for men in blue and women in pink. Age categories were compared by ANOVA analysis with Bonferroni *post hoc* testing, with the 35–44 years as reference, separately for men (blue asterisks) and women (pink asterisks). Additionally, the difference between men and women in the same age category is shown as black asterisks. *P*-values are indicated as *p < 0.05, **p < 0.01, ***p < 0.001, ****p < 0.0001.

**Figure 3:**
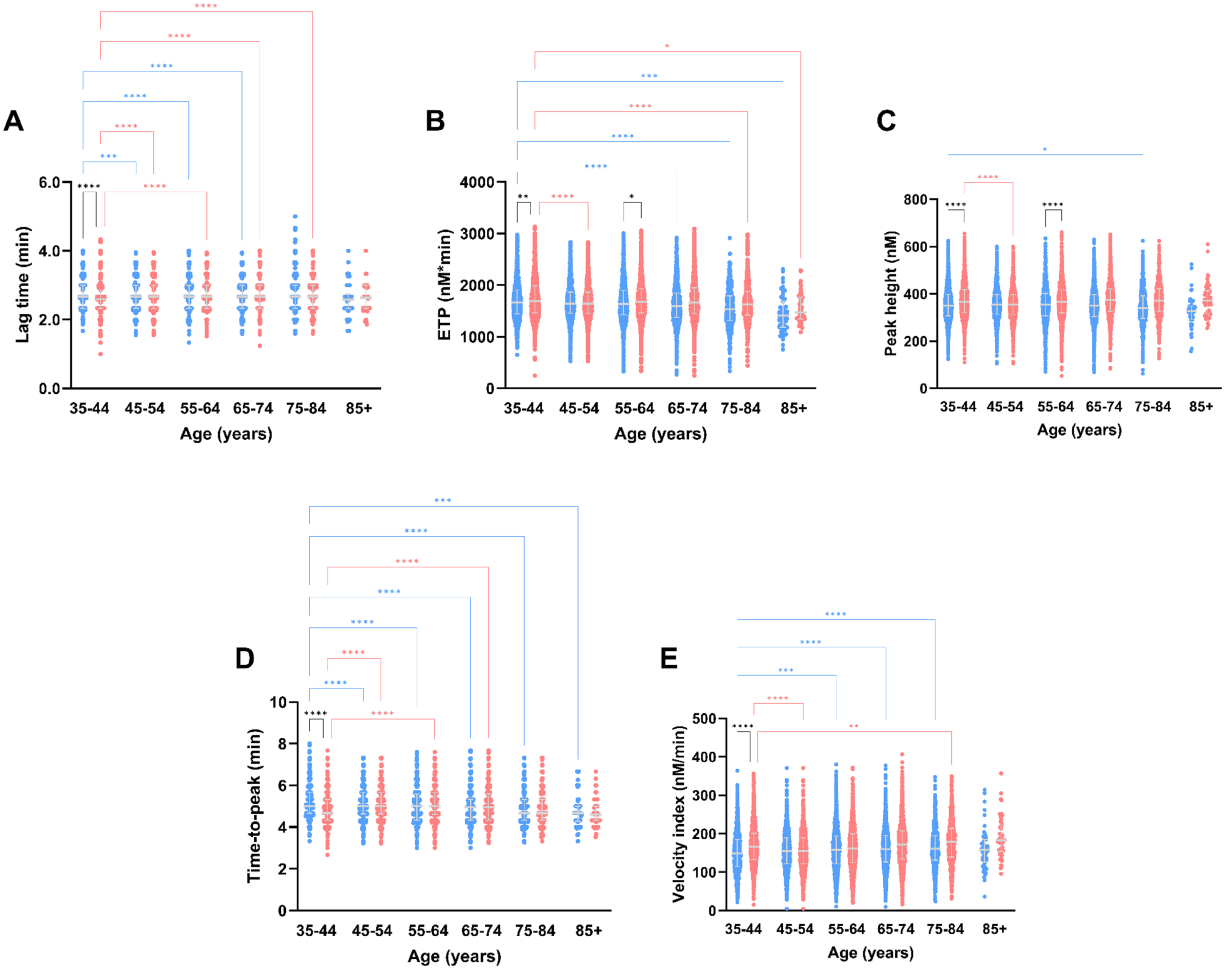
Thrombin generation parameters measured after stimulation with PPP reagent, stratified for age and sex. TG parameters lag time **(A)**, peak height **(B)**, time-to-peak **(C)**, ETP **(D)** and velocity index **(E)** according to the different age categories in the Moli-sani cohort. Data are depicted as the median and interquartile range. Data was stratified for sex, showing the data for men in blue and women in pink. Age categories were compared by ANOVA analysis with Bonferroni *post hoc* testing, with the 35–44 years as reference, separately for men (blue asterisks) and women (pink asterisks). Additionally, the difference between men and women in the same age category is shown as black asterisks. *P*-values are indicated as *p < 0.05, **p < 0.01, ***p < 0.001, ****p < 0.0001.

TG parameters differed between men and women in the same age category, and between age categories in both women and men (Figures 2, 3). The TG lag time is shorter in women than men at younger age (6%–7% lower in women), regardless of the trigger reagent used. In men, lag time shortens with increasing age, whereas women have a comparable or prolonged lag time at higher age, when PPP Reagent Low is used (Figure 2A). When a higher tissue factor stimulus is used (PPP Reagent), the lag time is shorter in men in higher age categories, whereas women show a prolongation of the lag time (Figure 3A, *P* < 0.001).

ETP is lower in men than in women, and regardless of the trigger reagent used (Figures 2B, 3B). Additionally, ETP decreases with increasing age in both men and women. The peak height is higher in women than men, independently of the trigger used (Figures 2C, 3C). Peak height increases with age in women but not in men (Figures 2C, 3C). Below 55 years of age, TTP is shorter in women than in men. Especially in men, TTP shortens with increasing age, independent of the tissue factor trigger used (Figures 2D, 3D). Velocity index increased with age, in both men and women, and regardless of the tissue factor trigger concentration used (Figures 2E, 3E).

### Effect of age and sex on the sensitivity of the activated protein C pathway

**Figure 4:**
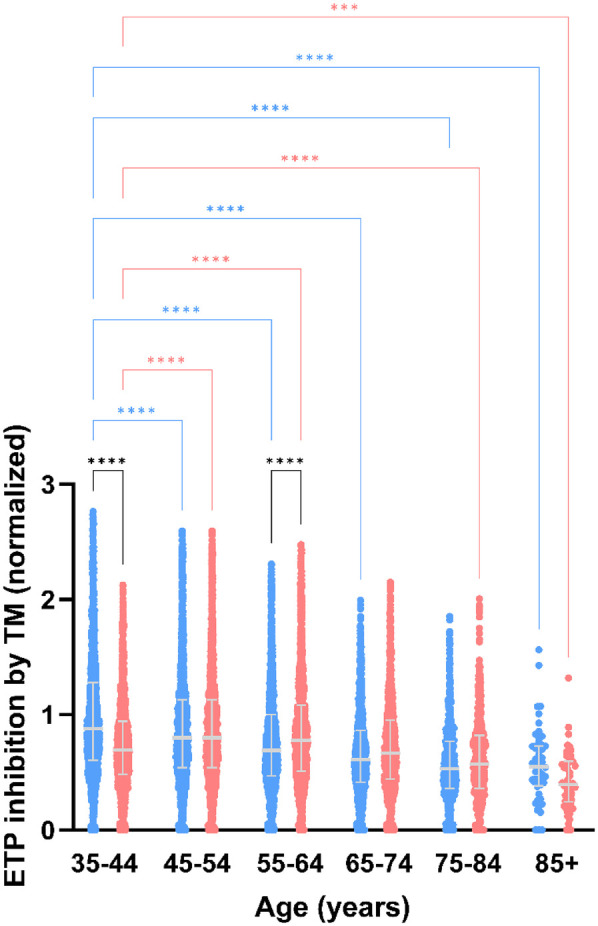
Thrombomodulin sensitivity of thrombin generation measured after stimulation with PPP reagent, stratified for age and sex. Normalized inhibition of ETP in men vs. women. Data are depicted as the median and interquartile range. Data was stratified for sex, showing the data for men in blue and women in pink. Age categories were compared by ANOVA analysis with Bonferroni *post hoc* testing, with the 35–44 years as reference, separately for men (blue asterisks) and women (pink asterisks). Additionally, the difference between men and women in the same age category is shown as black asterisks. *P*-values are indicated as *p < 0.05, **p < 0.01, ***p < 0.001, ****p < 0.0001.

The sensitivity of the activated protein C pathway was determined by comparing TG measured in the presence and absence of TM. The normalized inhibition of the ETP in the presence of TM was lower in men than in women, especially at younger age. Increasing age caused a desensitization of the APC pathway, in both men and women (Figure 4).

## Discussion

Hypercoagulability is more common in older than younger individuals, if no underlying genetic or acquired condition is present (37). Cardiovascular diseases, such as myocardial infarction or coronary heart disease, which are associated with hypercoagulability and thrombosis, are more prevalent in the elderly population (38). Additionally, there is a marked difference between hypercoagulability and cardiovascular disease between men and women (39). The TG test is a global test for hypercoagulability and has been shown increased in individuals with cardiovascular diseases (39). Nevertheless, no consensus had been reached on the effect of age on TG test parameters (19–23). Moreover, it had not yet been addressed whether the changes in TG with age are comparable for men and women, even though TG is known to differ substantially between men and women in general (26).

In this study, we show that the TG profile becomes more “procoagulant” with increasing age in a large subset of the general Italian adult population. The combination of a prolonged lag time, shortened time-to-peak, and elevated peak height show that the generation of thrombin takes place in a more explosive burst at older age compared to TG in younger subjects. Our results are in agreement with the results of Van Paridon et al. and Wu et al., who found an increased lag time in elderly subjects (19, 22). Moreover, Haidl et al. and Thangaranju et al. previously showed that older subjects have higher peak height and velocity index (21, 23).

Our study shows that men and women age behave in a vastly different manner in regard to hypercoagulability. Whereas absolute TG parameter values in men tend to be stable, TG parameters in women shift towards a more procoagulant TG profile in a remarkable way. In women, velocity index increased by 23% from the age of 41 until the age of 88, whereas in men, the velocity index increased only by 4%. Another well described difference in TG between men and women is the sensitivity of the APC pathway, especially in women using OC (40). In the current study, we investigated the sensitivity of the APC pathway by adding TM, which binds to thrombin and initiates the activation of the protein C mediated negative feedback loop (28, 31, 40–43). A desensitization of the APC pathway has been reported in women using OC, and has been postulated as one of the possible causes behind the increased risk of thrombosis associated with OC use (42, 44). Indeed, we found that in the age groups with the highest amount of oral contraceptive use, the activated protein C pathway was less sensitive to the inhibitory actions of thrombomodulin in women than in men. Moreover, with increasing age, the APC pathway becomes increasingly desensitized in both men and women. The latter could in part explain the higher risk of thrombotic events later in life (16). In older individuals, the use of vitamin K antagonists typically causes APC pathway desensitization due to the reduction of protein C levels (45). However, in this study, we excluded subjects using VKAs from analysis, because this vastly affects TG. Interestingly, TM shedding is known to increase with age as well, although soluble TM has been reported to be less active in the activation process of protein C than membrane bound TM (46, 47).

A causal explanation for the changing of TG parameters throughout life are the individual coagulation factors and sub-processes that underly the process of TG. Dielis et al. previously published that fibrinogen levels are positively associated with age (20). Fibrinogen is known to stimulate thrombin generation as it increases the peak height substantially, by shielding thrombin from inhibition by its natural inhibitors (48). Furthermore, lower antithrombin levels are associated with older age in this cohort (49) and others (20). Lower levels of antithrombin are known to result in higher TG peak height, due to the reduced capacity to inhibit thrombin (50). Moreover, the plasma levels of procoagulant factors such as FV, FVII, FVIII, FIX, and FX have been reported to increase with age (20). The increase in these procoagulant factors upstream in the generation of thrombin itself, might be a very reasonable explanation for an increased and faster production of thrombin (50). The outcome of the thrombin generation test is determined by the balance between prothrombin conversion and thrombin inactivation (51). Therefore it is likely that the changes in either prothrombin conversion or thrombin inactivation associated with age and/or sex are caused by multiple underlying changes. In preliminary analyses using the thrombin dynamics method we have found that both prothrombin conversion, and well as thrombin inactivation are reduced at older age (unpublished data) (50).

Our study emphasizes the need for age-specific reference ranges, or at least the opportunity to take age into consideration when interpreting TG results in patient populations. Both age and sex have a profound effect on TG parameters, and we show that TG parameter values considered normal for a certain age or sex category, might not be normal for another group of individuals. A limitation of our study is that it has been performed in a cohort of Caucasian, Italian individuals, and it yet has to be investigated whether our conclusions are comparable in other populations. Nevertheless, our findings are in line with those from several other populations, from different regions and ethnicity (19, 21–23). Another limitation of the study is that we were unable to measure all coagulation parameters of interest, due to the a small sample volume and the large sample size.

In conclusion, we found that the TG profile becomes more procoagulant at older age, and that this effect is more pronounced in women than men. The sensitivity of the APC pathway to TM decreases with increasing age in men and women. Therefore, age and sex appropriate reference values for TG parameters would be of interest for the ongoing clinical implementation of the TG test.

## Data Availability

The data underlying this article will be shared on reasonable request to the corresponding author. The data are stored in an institutional repository (https://repository.neuromed.it/index.php/login) and access is restricted by the ethical approvals and the legislation of the European Union.

## Group members of Moli-sani investigators

The enrolment phase of the Moli-sani Study was conducted at the Research Laboratories of the Catholic University in Campobasso (Italy), the follow up of the Moli-sani cohort is being conducted at the Department of Epidemiology and Prevention of the IRCCS Neuromed, Pozzilli, Italy.

**Steering Committee:** Licia Iacoviello*#(Chairperson), Giovanni de Gaetano*, Maria Benedetta Donati*.

**Scientific Secretariat:** Chiara Cerletti* (Coordinator), Marialaura Bonaccio*, Americo Bonanni*, Simona Costanzo*, Amalia De Curtis*, Augusto Di Castelnuovo*, Alessandro Gialluisi*#, Francesco Gianfagna§°, Mariarosaria Persichillo*, Teresa Di Prospero* (Secretary).

**Safety and Ethical Committee:** Jos Vermylen (Catholic University, Leuven, Belgio) (Chairperson), Renzo Pegoraro (Pontificia Accademia per la Vita, Roma, Italy), Antonio Spagnolo (Catholic University, Roma, Italy).

External Event Adjudicating Committee: Deodato Assanelli (Brescia, Italy), Livia Rago (Campobasso, Italy).

**Baseline and Follow-up Data Management:** Simona Costanzo*(Coordinator), Marco Olivieri (Campobasso, Italy), Sabatino Orlandi*, Teresa Panzera*.

**Data Analysis:** Augusto Di Castelnuovo* (Coordinator), Marialaura Bonaccio*, Simona Costanzo*, Simona Esposito*, Alessandro Gialluisi*#, Anwal Ghulam*, Francesco Gianfagna§°, Antonietta Pepe*, Emilia Ruggiero*, Sukshma Sharma*.

**Biobank, Molecular and Genetic Laboratory:** Amalia De Curtis* (Coordinator), Concetta Civitillo*, Alisia Cretella*, Sara Magnacca*, Fabrizia Noro*.

**Recruitment Staff:** Mariarosaria Persichillo* (Coordinator), Francesca Bracone*, Fiorella De Rita (Cuore Sano ETS), Giuseppe Di Costanzo*, Sabrina Franciosa*, Martina Morelli*.

**Communication and Press Office:** Americo Bonanni*.

**Regional Institutions:** Direzione Generale per la Salute-Regione Molise; Azienda Sanitaria Regionale del Molise (ASReM, Italy); Agenzia Regionale per la Protezione Ambientale del Molise (ARPA Molise, Italy); Molise Dati Spa (Campobasso, Italy); Offices of vital statistics of the Molise region.

**Hospitals:** Presidi Ospedalieri ASReM: Ospedale A. Cardarelli-Campobasso, Ospedale F. Veneziale-Isernia, Ospedale San Timoteo-Termoli (CB), Ospedale Ss. Rosario-Venafro (IS), Ospedale Vietri-Larino (CB), Ospedale San Francesco Caracciolo-Agnone (IS); Casa di Cura Villa Maria-Campobasso; Responsible Research Hospital-Campobasso; IRCCS Neuromed-Pozzilli (IS).

^***^ *Research Unit of Epidemiology and Prevention, IRCCS Neuromed, Pozzilli, Italy*

^*#*^ *Department of Medicine and Surgery, LUM University “Giuseppe Degennaro”, Casamassima, Italy*

^*§*^ *Mediterranea Cardiocentro, Napoli, Italy*

*°Department of Medicine and Surgery, University of Insubria, Varese, Italy*

*Moli-sani Study Past Investigators are available at* https://www.moli-sani.org/?page_id=173
